# Commissioning and clinical evaluation of the IDENTIFY^TM^ surface imaging system for frameless stereotactic radiosurgery

**DOI:** 10.1002/acm2.14058

**Published:** 2023-06-08

**Authors:** Elizabeth L. Covington, Dennis N. Stanley, Rodney J. Sullivan, Kristen O. Riley, John B. Fiveash, Richard A. Popple

**Affiliations:** ^1^ Department of Radiation Oncology University of Alabama at Birmingham Birmingham Alabama USA; ^2^ Department of Radiation Oncology University of Michigan Ann Arbor Michigan USA; ^3^ Department of Neurosurgery University of Alabama at Birmingham Birmingham Alabama USA

**Keywords:** optical surface imaging, SGRT, SRS, stereotactic radiosurgery, surface guided radiation therapy

## Abstract

**Purpose:**

To commission and assess the clinical performance of a new commercial surface imaging (SI) system by analyzing intra‐fraction motion from the initial cohort of patients treated with frameless stereotactic radiosurgery (fSRS).

**Methods:**

The IDENTIFY^TM^ SI system was commissioned for clinical use on an Edge (Varian Medical Systems, Palo Alto, CA) linear accelerator. All patients who received intracranial radiotherapy with HyperArc^TM^ (Varian Medical Systems, Palo Alto, CA) were immobilized with the Encompass^TM^ (Qfix, Avondale, PA) thermoplastic mask and monitored for intra‐fraction motion with SI. IDENTIFY^TM^ log files were correlated with trajectory log files to correlate treatment parameters with SI‐reported offsets. IDENTIFY^TM^ reported offsets were correlated with gantry and couch angles to assess system performance for obstructed and clear camera field of view. Data were stratified by race to evaluate performance differences due to skin tone.

**Results:**

All commissioning data were found to meet recommended tolerances. IDENTIFY^TM^ was used to monitor intra‐fraction motion on 1164 fractions from 386 patients. The median magnitude of translational SI reported offsets at the end of treatment was 0.27 mm. SI reported offsets were shown to increase when camera pods are blocked by the gantry with larger increases seen at non‐zero couch angles. With camera obstruction, the median magnitude of the SI reported offset was 0.50 and 0.80 mm for White and Black patients, respectively.

**Conclusions:**

IDENTIFY^TM^ performance during fSRS is comparable to other commercially available SI systems where offsets are shown to increase at non‐zero couch angles and during camera pod blockage.

## 1 INTRODUCTION

Surface‐guided radiotherapy (SGRT) utilizes surface imaging (SI) systems to optically monitor the surface of a patient for assistance with patient set‐up,^1^, ^2^ respiratory gating,^3^, ^4^ and intra‐fraction motion management.^5^, ^6^, ^7^, ^8^ During frameless stereotactic radiosurgery (fSRS), an open‐face thermoplastic mask is used to immobilize patients while allowing for visualization of a tracking surface by the SI system. SI for fSRS is less invasive than frame‐based SRS while maintaining the sub‐millimeter tracking of intra‐fraction motion.^5^, ^6^, ^9^

Due to the use of small or no margins in fSRS treatment planning, single isocenter treatment planning, and single fraction delivery, having sub‐millimeter performance across all clinical indications is of the utmost importance for intra‐fraction motion management during fSRS.^10^, ^11^ SI has been previously reported to suffer from suboptimal performance at non‐zero couch angles; therefore, thorough characterization of clinical performance at a variety of gantry and couch angles is recommended during commissioning.^5^, ^11^, ^12^ New professional guidance has also been recently published for recommendations for commissioning and performing routine quality assurance on SI systems in AAPM task group report 302: SGRT.^13^

In this work, we detail the commissioning and initial clinical experience with IDENTIFY™ (Varian Medical System, Palo Alto, CA). IDENTIFY™ is a SI system that consists of three stereoscopic camera pods separated by approximately 90 degrees. Each pod projects a speckle pattern that is used to reconstruct camera images into a 3D surface. Further technical details of the IDENTIFY™ system have been reported elsewhere.^13^, ^14^ At our institution, IDENTIFY™ system is installed in a vault with an Edge (Varian Medical Systems, Palo Alto, CA) and is used for monitoring intra‐fraction motion during intracranial stereotactic radiosurgery and stereotactic radiotherapy. As a new product and relatively new technology, commissioning and longitudinal quality data and intra‐fraction patient motion data are provided to assist physicists in understanding the performance and limitations of IDENTIFY™ in the context of stereotactic radiosurgery intra‐fraction motion monitoring.

## 2 METHODS AND MATERIALS

The IDENTIFY^TM^ system software was version 2, and the results reported here span releases 2.1, 2.2, and 2.3.

### 2.1 Commissioning

To commission the IDENTIFY™ system, tests were performed to assess the thermal drift, field of view, and difference in SI‐reported offsets as a function of isocenter location. To assess the thermal drift of the system, the QUASAR™ Penta‐Guide Phantom (Modus Medical Devices, Ontario, Canada) was monitored by selecting the entire surface as the region of interest (ROI) and recording offsets after reference capture. The field of view was assessed by shifting the same phantom along each translational axis until IDENTIFY™ could no longer track the phantom.

To test the accuracy of offsets, the Penta‐Guide phantom was monitored during couch movement. Per TG‐302, localization should be tested over a reasonable clinical range, defined as ±10 cm range from isocenter. After allowing the system to warm up for approximately 15 min, a reference surface of the Penta‐Guide phantom was captured. Known offsets in the range of ±10 cm were applied along each axis via couch motions. Since expected motion during SRS is expected to be much smaller, localization accuracy was also tested over ±0.5 cm from isocenter. To evaluate the accuracy over the range ±0.5 cm, an anthropomorphic foam head, with an ROI similar to that to be used for patients, was monitored while applying known randomly selected offsets. We explored two methods for applying known offsets. First, we used the micrometer‐driven translation stage provided with the Varian cone package that is intended to position a Winston‐Lutz pointer. This method is very accurate, providing positioning accuracy of <0.1 mm. Note that this method requires using a foam phantom because the translation stage is not able to support the weight of a plastic anthropomorphic phantom. Furthermore, this method can only apply translations. The second method applied offsets by moving the treatment couch. This method is simple, but couch positioning error contributes uncertainty to the applied offset. Couch‐applied motion also allows for testing rotational accuracy.

SI has been previously reported to suffer from reduced accuracy at non‐zero couch angles; therefore, we used a previously reported method for quantifying the IDENTIFY™ system's residual error at non‐zero table angles.^15^ Briefly, the test is based on the evaluation of couch walkout using a radiopaque ball bearing (BB). A tungsten carbide BB was embedded in a polystyrene head phantom to simultaneously collect the phantom's position with MV imaging and SI. The BB is positioned at isocenter using orthogonal kV images obtained by the onboard image guidance system. At multiple couch angles, MV images are obtained and used to determine the lateral and longitudinal position as the couch is rotated. Note that any deviations from isocenter are attributed to couch walkout. Simultaneously, the offsets reported by the IDENTIFY™ system are recorded. The differences between the EPID and the IDENTIFY™ offsets give the residual error of the IDENTIFY™ system. The residual SGRT error (RSE) was calculated using Equation (1)

(1) $$RSE\;=\sqrt{{\left(Lat_{MV}-Lat_{SI}\right)}^{2}+{\left(Lng_{MV}-Lng_{SI}\right)}^{2}}$$

where Lat and Lng are the lateral and longitudinal positions as determined by MV and SI systems respectively. We repeated this test with the BB at different depths using a procedure that has been previously reported.^15^

An end‐to‐end test was performed by CT scanning an anthropomorphic head phantom (Stereotactic End‐to‐End Verification Phantom [STEEV], CIRS, Norfolk) in the Encompass™ mask system (QFix, Avondale, PA), importing it into the treatment planning system, and creating a representative SRS plan. The SI region‐of‐interest was created in the treatment planning system, a 5 mm uniform retraction off the edge of the open region of the mask, and exported to the IDENTIFY™ system. The phantom was then aligned via CBCT following the clinical SRS imaging protocol and reported offsets after radiographic alignment were recorded.

Monthly QA was performed following the vendor recommendations which includes taking a reference CT scan that is used as the reference image for CBCT alignment during QA. To facilitate the alignment of the phantom, we contoured the spherical air cavities to use for auto‐matching. Immediately before QA, the Penta‐Guide phantom was placed on the couch and monitored for 10−15 min to minimize spatial drift. A CBCT was performed using a 1 mm slice thickness and aligned with auto‐matching using an ROI of the contoured spherical air cavities with a 1.0 cm margin.

### 2.2 Clinical data

**FIGURE 1:**
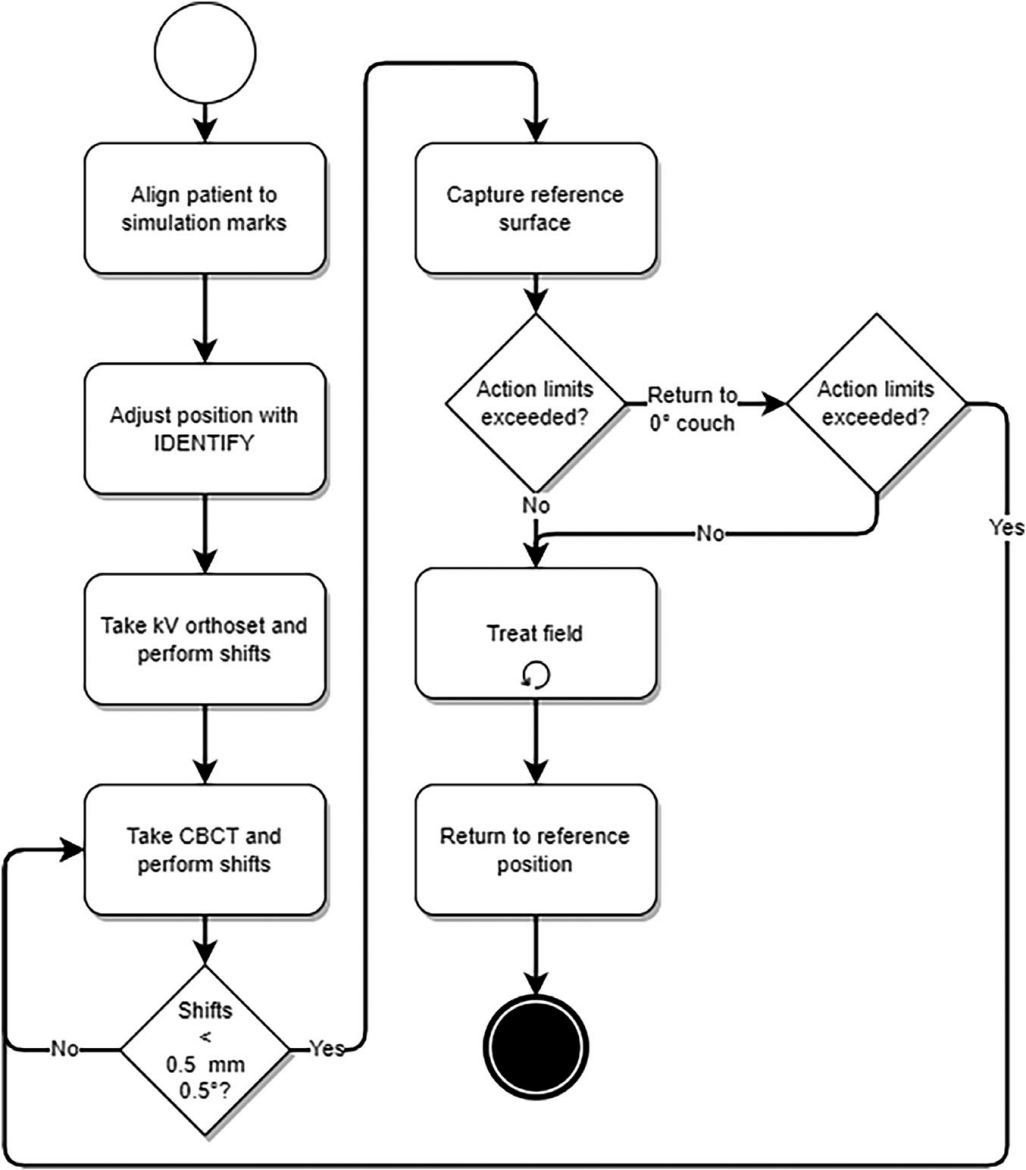
Clinical workflow for using IDENTIFY™ to monitor intra‐fraction motion during SRS/SRT.

Patients were simulated with an Encompass™ (Qfix, Avondale, PA) thermoplastic mask. The body and the open‐face regions were contoured in the Eclipse (Varian Medical Systems, Palo Alto, CA) treatment planning system, exported to IDENTIFY™, and set as the reference surface and ROI, respectively. The ROIs were contoured following the procedure described in Covington et al.^5^ with two modifications. First, because the edge of the open‐face region of the Encompass™ mask was readily identified on the treatment planning CT, it was not marked with radiopaque wire. Second, per vendor recommendation, the eyes were included in the ROI. The ROI was not further modified in the IDENTIFY™ system. The IDENTIFY™ workflow for SRS is shown in Figure 1. Monitoring with IDENTIFY™ was performed during treatment set‐up, and patients' heads were adjusted to minimize rotations before securing the top portion of the mask. During the initial setup, prior to image acquisition, logging of the IDENTIFY reported offsets to a file was manually initiated. Once the initial alignment was performed with SI, an orthogonal kV pair followed by CBCT was performed for alignment. Upon completion of the radiographic alignment, a new reference surface was taken in IDENTIFY™ to zero out all translational and rotational offsets, and treatment was initiated. If SI‐reported offsets exceed tolerances during treatment, patients were returned to a neutral couch position. If tolerances were still exceeded, radiographic imaging was repeated for realignment, a new reference surface was acquired, and treatment was resumed.

After treatment, SI logs containing timestamps with intra‐fraction motion data in all translational and rotational directions were combined with linear accelerator trajectory logs to correlate SI‐reported offsets with gantry rotation and couch angle. To synchronize the SI and trajectory logs, the difference between the IDENTIFY™ and treatment console clocks was monitored and used to correct the timestamps. For each beam, the following data points were obtained: (1) at beam‐on (gantry either 0 or 180) where the gantry did not block any camera pods, (2) during beam‐on where the gantry did not block any camera pods (gantry 140 or 220), or (3) during beam‐on with camera blocked, where one camera pod was fully obscured by gantry motion (gantry 50 or 310).

To validate SI system performance for different patient skin tones, the oncology information system (OIS) was used to identify the patient's race. Patients were classified into the following groups per ARIA: White, Black, or not‐specified (NS). To confirm that race was an appropriate surrogate for SI skin tone setting, we randomly selected 20 patients, 10 White and 10 Black, randomly shuffled the patient identifier, and three of the authors independently assessed the face photo in Aria and classified the skin tone as light or dark.

Patient data were collected and analyzed as part of a project approved by The University of Alabama Institutional Review Board (IRB‐080613002).

## 3 RESULTS

### 3.1 Commissioning

**FIGURE 2:**
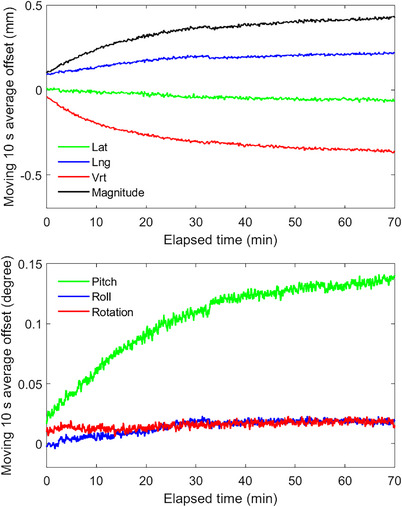
Spatial drift measured from IDENTIFY™ for a stationary phantom.

To quantify thermal drift, the Penta‐Guide phantom was monitored for 70 min from a cold‐camera state. Figure 2 shows the vertical (Vrt), longitudinal (Lng), lateral (Lat), and translation magnitude, and the pitch, roll, and rotation SI reported offsets over this period.

**FIGURE 3:**
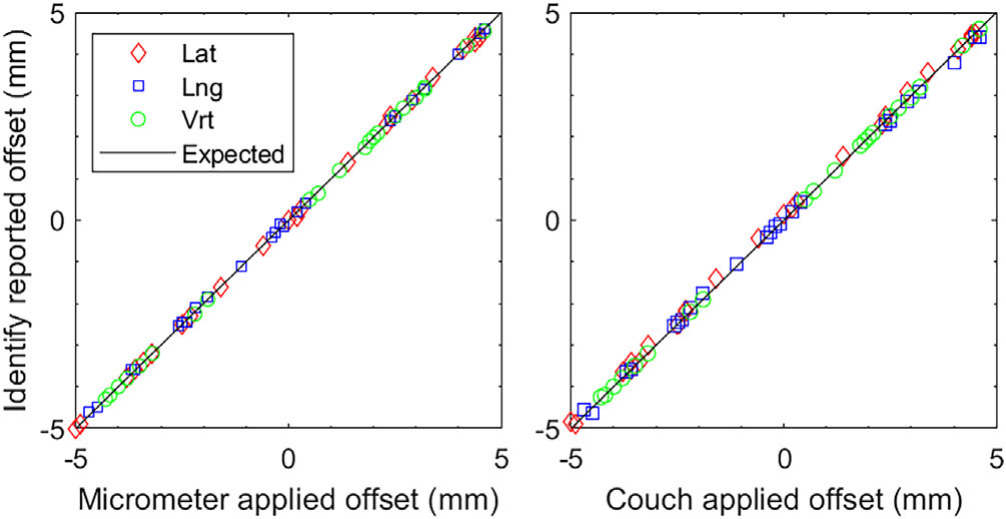
IDENTIFY™ reported offset of lateral (Lat), longitudinal (Lng), and vertical (Vrt) motion versus (left) micrometer‐applied offset and (right) couch‐applied offset (without rotation) on an anthropomorphic phantom with an SRS representative region of interest.

For translations applied using the treatment table over the range ±10 cm, each axis was evaluated independently. The largest error was 0.3 mm. Figure 3 and Table 1 show the IDENTIFY™ reported offset for translations and rotations applied in the range ±0.5 cm in all translational directions versus the micrometer and couch applied offsets.

**TABLE 1:** The mean difference in the applied translational offset, performed by either micrometer or couch movement, and the IDENTIFY™ reported offset.

	Mean difference (standard deviation) between applied and IDENTIFY™ offsets					
	Vrt (mm)	Lng (mm)	Lat (mm)	Rot (deg)	Pitch (deg)	Roll (deg)
Translation applied with micrometer	0.01 (0.02)	−0.02 (0.05)	0.01 (0.04)	–	–	–
Translation applied with couch	0.00 (0.01)	0.01 (0.10)	−0.10 (0.08)	–	–	–
Translation and rotation applied with couch	−0.00 (0.06)	0.00 (0.13)	−0.09 (0.15)	−0.17 (0.48)	−0.08 (0.33)	0.03 (0.35)

**FIGURE 4:**
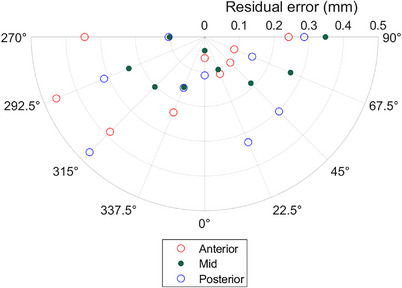
The IDENTIFY™ residual SGRT error (RSE), the magnitude of the difference between the MV imaging and IDENTIFY™ reported offsets (see Equation 1), as a function of table angle for different isocenter locations within an anthropomorphic phantom.

Figure 4 shows the IDENTIFY^TM^ residual error for isocenter placed directly at the surface of the phantom (anterior), mid‐depth (mid), and posterior surface (posterior).

For end‐to‐end testing using an anthropomorphic phantom, the magnitude of IDENTIFY^TM^ reported translational offsets was 0.23 mm after CBCT alignment.

### 3.2 QA results

**FIGURE 5:**
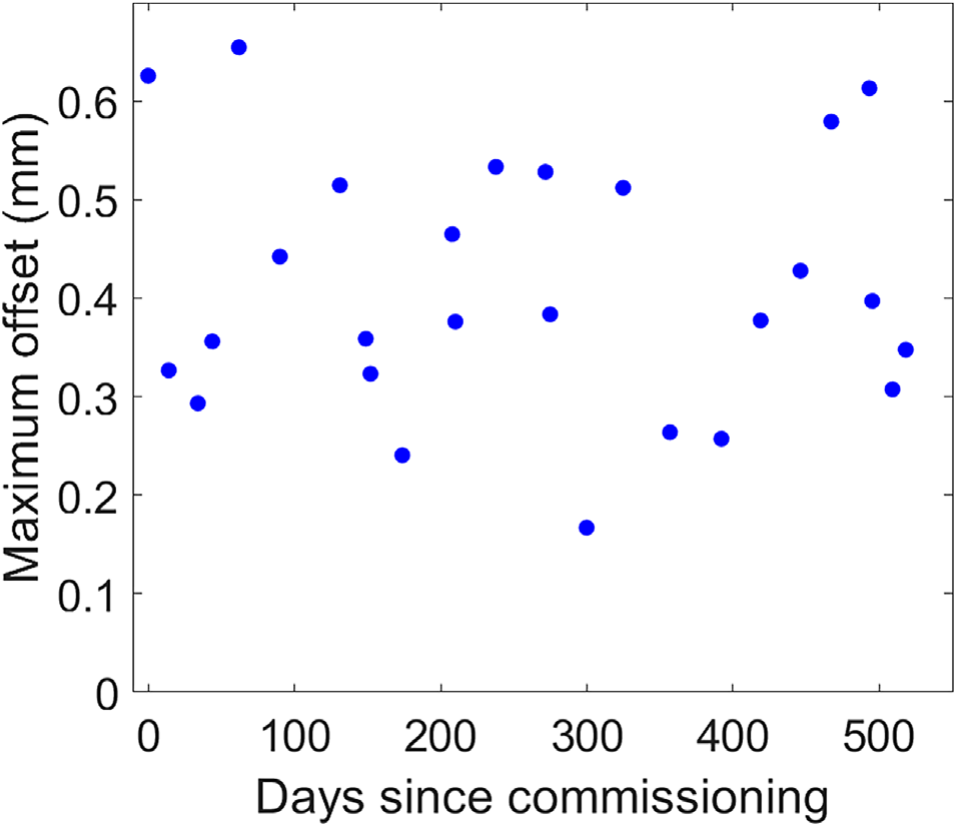
The maximum IDENTIFY™ residual SGRT error (RSE) out of all couch angles tested during monthly QA as a function of days since commissioning.

The localization accuracy at non‐zero table angles was tested at least monthly using the procedure previously reported.^15^ The maximum difference between IDENTIFY™ and the EPID position for The University of Alabama at Birmingham system since commissioning is shown in Figure 5.

### 3.3 Patient data

Monitoring was performed on 1164 fractions of HyperArc™ from 386 patients spanning 457 days from March 2021 to June 2022. Of these 1164 fractions, 93 were omitted from analysis due to the following: 32 were missing the IDENTIFY™ data file, 17 were missing the trajectory log, 20 had incomplete trajectory logs, and 24 could not be due to mid‐treatment imaging which required the recapture of the SI reference surface. The average time from the first beam‐on to the last beam‐off was 3.35 min (range 1.87–7.45 min). Since patients were treated with Hyperarc, couch angles were limited to 0, 45, 90, 315, and 270 degrees.

The median magnitude of SI reported offset from the beginning to end of treatment was 0.27 mm (interquartile range [IQR] 0.17–0.42 mm). Table 2 shows the median magnitude and IQR of translational SI‐reported offsets before beam‐on at non‐zero couch angles and the end of treatment. Table 3 shows the median magnitude and IQR of translational SI‐reported offsets before beam‐on at non‐zero couch angles when the camera pods are clear or blocked.

**TABLE 2:** The mean and interquartile range (IQR) of IDENTIFY™ reported offsets before beam‐on at non‐zero couch angles.

Couch angle (°)	Vertical (IQR) mm	Longitudinal (IQR) (mm)	Lateral (IQR) (mm)
270	−0.23 (0.11)	−0.64 (0.61)	0.10 (0.27)
225	−0.11 (0.20)	−0.41 (0.51)	−0.36 (0.29)
135	−0.08 (0.20)	−0.20 (0.48)	−0.21 (0.25)
90	−0.17 (0.27)	−0.05 (0.47)	−0.10 (0.26)
0° (End of treatment)	0.03 (0.15)	−0.03 (0.32)	0.00 (0.14)

**TABLE 3:** The median magnitude (MAG) and interquartile range (IQR) of IDENTIFY™ reported offsets at non‐zero couch angles when the camera is clear or blocked.

	MAG (IQR) mm	
Couch angle (°)	Camera clear	Camera blocked
270	0.40 (0.32)	0.50 (0.26)
225	0.38 (0.26)	0.47 (0.41)
180	0.16 (0.15)	0.22 (0.20)
135	0.55 (0.24)	0.60 (0.34)
0	0.48 (0.33)	0.45 (0.29)

**FIGURE 6:**
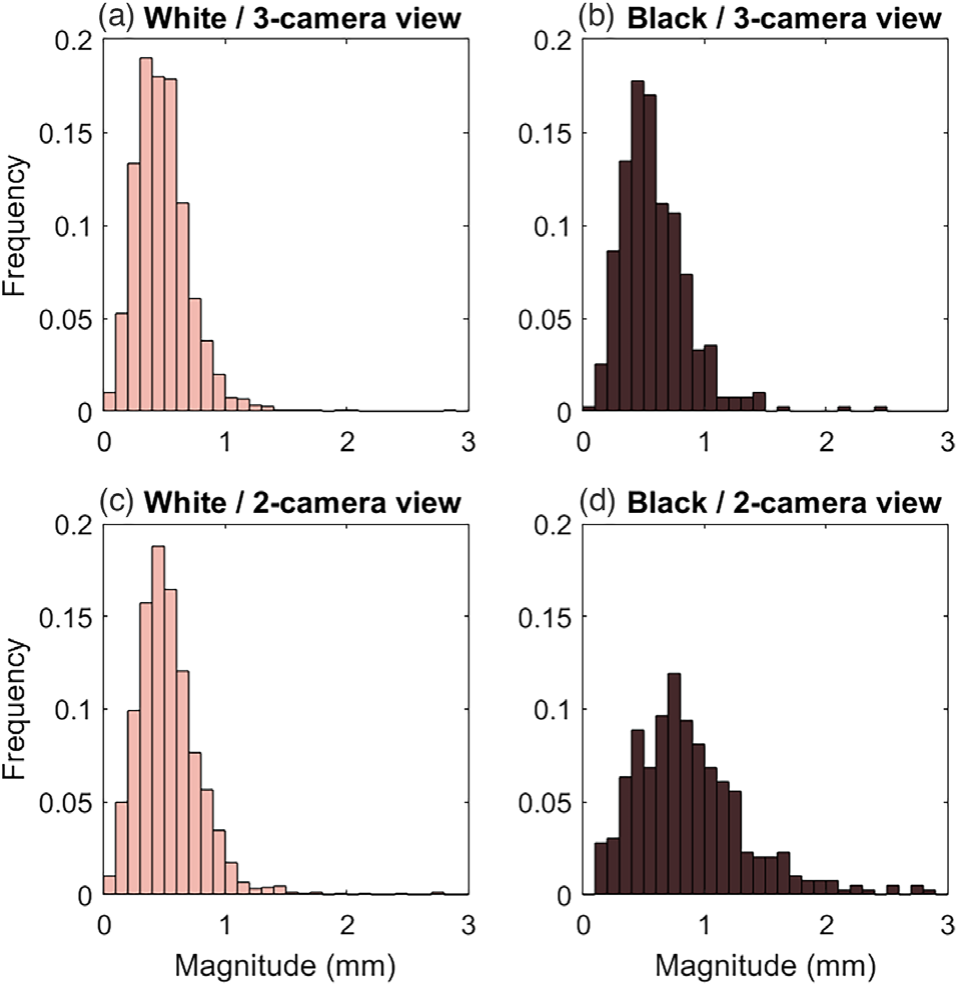
Frequency of IDENTIFY™ reported translational magnitude at non‐zero table angles for (a) White patients with no cameras obstructed, (b) Black patients with no cameras obstructed, (c) White patients with one camera obscured, and (d) Black patients with one camera obstructed.

Of the 370 patients in the evaluated data, 258 (69.7%) were White, and 79 (21.4%) were Black. For the 20 patients selected to check the correlation between race and skin tone, all three evaluators classified all 10 White patients as having light skin tone and all 10 Black patients as having dark skin tone. Frequency histograms of the offsets reported at non‐coplanar table angles as a function of patient race and camera obstruction are shown in Figure 6. Without camera obstruction, the median magnitude was 0.46 and 0.55 mm for White and Black patients, respectively. With camera obstruction, the median magnitude was 0.50 and 0.80 mm for White and Black patients, respectively. The Wilcoxon rank sum test *p*‐values were <0.001. Data were evaluated to quantify the number of treatment fractions where the IDENTIFY™ reported magnitude exceeded 1.0 mm. Table 4 shows the median magnitudes and the percentage of treatment fractions where IDENTIFY™ magnitudes exceed 1.0 mm stratified by race and the number of cameras.

**TABLE 4:** Median offset magnitude and percent of treatment fractions with magnitudes exceeding 1 mm. All comparisons between median values had *p* < 0.001 for the two‐sided Wilcoxon rank sum test. The 95% confidence interval (CI) for the fraction of magnitudes exceeding 1 mm was calculated using the Clopper‐Pearson method to calculate confidence intervals for a binomial distribution.

		3‐camera view		2‐camera view	
		Median magnitude (mm)	Fraction > 1 mm (95% confidence interval)	Median magnitude (mm)	Fraction > 1 mm (95% confidence interval)
Coplanar	White	0.15	0.4% (5/1256) (CI 0.1%−0.9%)	0.21	0.6% (7/1256) (CI 0.2%−1.1%)
	Black	0.18	0.6% (2/344) (CI 0.1%−2.1%)	0.28	0.9% (3/344) (CI 0.2%−2.5%)
Non‐coplanar	White	0.46	2.5% (38/1501) (CI 1.8%−3.5%)	0.50	4.3% (64/1501) (CI 3.3%−5.4%)
	Black	0.55	7.9% (31/394) (CI 5.4%−11.0%)	0.80	33.0% (130/394) (CI 28.4%−37.9%)

**FIGURE 7:**
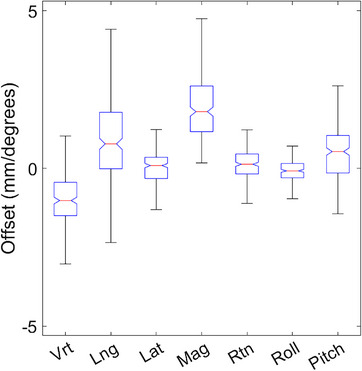
Difference between the IDENTIFY™ reported offsets utilizing the DICOM reference surface and the IGRT recorded shifts.

Starting in version 2.2 of the software, the reference surface source was included in the registration data file. This allowed evaluation of the offsets relative to the surface generated from the DICOM external contours immediately before the capture of the reference surface after image guidance. These offsets indicate how accurate the DICOM external surface is for setting up the patient. The median offset magnitude relative to the DICOM surface was 1.8 mm. Figure 7 shows box whisker plots for all translational and rotational differences from the DICOM reference surface and the IGRT recorded offsets from the first day of treatment.

## 4 DISCUSSION

Commissioning tests were chosen based on vendor recommendations and AAPM Task Groups 147 and 302. All data met vendor and task group recommended tolerances. Similar to other SI systems, IDENTIFY™ shows drift in reported offsets attributed to a lack of thermal equilibrium where the largest component of drift is in the vertical direction.^6^, ^11^ While the drift in magnitude was less than 0.5 mm over an hour, we recommend ensuring thermal stability before utilizing SI for intra‐fraction motion monitoring to not confuse thermal drift for patient motion. Based on the data shown in Figure 2, we ensure that the projectors have been on for at least 10 min before capturing a reference surface and starting treatment. This ensures that thermal drift will be less than 0.1 mm over the subsequent 10 min. Though the commissioning data in this study shows the technical performance of IDENTIFY™ is consistent with other commercial imaging systems, the data presented in this work shows the performance of a single system and does not necessarily reflect the performance of other systems. While this data can be used for comparison, each system should be tested and validated prior to clinical use.

While AAPM Task Groups 147 and 302 do not provide tolerances for the rotational accuracy of SGRT systems, we recommend evaluating the rotational accuracy during commissioning to inform clinical action limits. In the present work, we described the use of the 6‐degree‐of‐freedom couch to evaluate accuracy without significant couch rotation. Subsequent to commissioning, we developed a methodology for the evaluation of SGRT system performance in all 6 degrees of freedom over the entire range of couch rotation using linear accelerator automation. This methodology and the results obtained for the IDENTIFY™ system are reported elsewhere.^16^

Like other commercial SI systems, IDENTIFY™ suffers from decreased accuracy at non‐zero couch angles as shown in Table 2. We note that the largest source of residual error is in the longitudinal direction as previously reported with a different SI system.^12^ The largest discrepancy in the DICOM reference surface was also found to be in the longitudinal direction. This indicates that SI systems suffer from decreased localization accuracy in the longitudinal direction which may be attributed to the camera configuration. Due to the difference between the radiographic image alignment reported shifts and IDENTIFY™ reported offsets, we do not recommend using SI for initial patient alignment or realignment during treatment. Radiographic imaging should continue to be the gold standard for patient alignment after which the SI reference should be captured.

One limitation of this study is that it only includes data analysis from patients with a single reference capture. When patients have confirmed motion during treatment with a CBCT, a new reference is captured which causes a discontinuity in tracking offsets from the beginning to the end of treatment. In this study, only 2% of fractions (24/1164) had CBCT confirmed intra‐fraction motion that required acquiring a new reference surface. This is comparable to a previous study where 2.2% of patients had CBCT confirmed intra‐fraction motion.^12^

During our initial use of the IDENTIFY™ system, we observed significant false excursions when a camera pod was obstructed by the gantry in a subpopulation of patients. These excursions were much larger than observed in phantom tests. We noted several factors that seemed to contribute to false excursions. First, we noted false excursions only when the table was rotated. Secondly, false excursions seemed more likely to occur when the view of one of the camera pods was obstructed. Finally, dark skin tone seemed to be a contributing factor. As shown in Table 4, the fraction of deviations greater than 1.0 mm was significantly larger for Black patients than for White patients. In a previous study evaluating the effect of skin tone with the performance of the AlignRT (VisionRT, London, UK) system, we did not see a significant difference in performance at non‐zero couch angles before beam‐on but the data were not analyzed during camera pod obstruction where we noticed the largest disparities for IDENTIFY™. The present data cannot be used to make a direct comparison between vendors. Many factors such as illumination, camera exposure, camera location, image processing parameters, and so forth, may contribute to performance differences between SI systems. TG‐302 recommends that “the effect of surface color on localization accuracy should be assessed by testing both light‐ and dark‐toned phantoms when possible.” We used patient data instead of phantom testing due to a lack of access to appropriate phantoms having realistic skin tones. The utilization of anthropomorphic phantoms covering the spectrum of skin tones for SI commissioning and routine QA is currently under investigation.

Another limitation of this study is that data are restricted to one system and may not reflect the behavior of all IDENTIFY™ systems. While the system was installed according to vendor specifications, there may be variations in environmental factors such as ambient lighting or temperature fluctuations that could contribute to differences in performance between systems. Due to the decrease in localization accuracy with non‐zero couch angles and camera pod obstruction during gantry motion, it is important to thoroughly characterize the system performance at a variety of clinically relevant couch and gantry angles and include this data while setting action levels for halting treatment. Note that a portion of the offsets recorded by SI at non‐zero couch angles are expected due to couch walkout and must also be considered. We recommend including three factors when setting the action levels: (1) SI residual error, (2) couch walk‐out, and (3) spatial drift.

## 5 CONCLUSIONS

Commissioning and infraction motion data for 1164 SRS treatments for IDENTIFY^TM^ shows comparable system performance to other commercial SI systems. The commissioning data demonstrated accuracy <0.1 mm at table angle zero and <0.5 mm at non‐zero table angles. Like other SI systems, localization accuracy decreases at non‐zero couch angles and when camera pods are obstructed by gantry motion. In clinical use, a small but non‐negligible false excursion rate correlated with camera obstruction and dark skin tone has been observed that must be accounted for in clinical decision‐making.

## AUTHOR CONTRIBUTIONS

All authors made substantial contributions to the conception and design of the work; the acquisition, analysis, and interpretation of data for the work; drafting the work and revising it critically for important intellectual content; gave final approval of the version to be published; and agrees to be accountable for all aspects of the work in ensuring that questions related to the accuracy or integrity of any part of the work are appropriately investigated and resolved.

## CONFLICT OF INTEREST STATEMENT

This work was funded by Varian Medical Systems.
