# Correction: “Commissioning and clinical evaluation of the IDENTIFYTM surface imaging system for frameless stereotactic radiosurgery” https://doi.org/10.1002/acm2.14058

**DOI:** 10.1002/acm2.14444

**Published:** 2024-08-06

**Authors:** Elizabeth L. Covington, Dennis N. Stanley, Rodney J. Sullivan, Kristen O. Riley, John B. Fiveash, Richard A. Popple

**Affiliations:** ^1^ University of Michigan Ann Arbor Michigan USA; ^2^ University of Alabama at Birmingham Birmingham Alabama USA

An incorrect table was inadvertently uploaded into the original manuscript. The below table contains the correct values for the median and interquartile range values for the IDENTIFY™ surface imaging system before beam‐on at non‐zero couch angles.

**TABLE 2:** Median and interquartile range (IQR) of IDENTIFY™ reported offsets before beam‐on at non‐zero couch angles.

Couch angle (°)	Vertical (IQR) mm	Longitudinal (IQR) (mm)	Lateral (IQR) (mm)
270	−0.01 (0.47)	−0.03 (0.33)	0.16 (0.32)
225	−0.06 (0.17)	0.21 (0.38)	−0.16 (0.18)
135	−0.08 (0.16)	0.19 (0.38)	−0.41 (0.19)
90	−0.14 (0.20)	0.05 (0.54)	−0.25 (0.24)
0° (end of treatment)	−0.05 (0.18)	−0.03 (0.35)	−0.01 (0.15)

